# Single antiplatelet regimen in flow diverter treatment of cerebral aneurysms: The drug matters. A systematic review and meta-analysis

**DOI:** 10.1177/15910199231177745

**Published:** 2023-05-23

**Authors:** Yihui Ma, Jawid Madjidyar, Tilman Schubert, Patrick Thurner, Isabelle Barnaure, Zsolt Kulcsar

**Affiliations:** 1 Department of Neurosurgery, 89674 Zhongnan Hospital of Wuhan University, Wuhan, China; 2 Department of Neuroradiology, Clinical Neuroscience Center, 27243 University Hospital Zurich, University of Zurich, Zurich, Switzerland

**Keywords:** Flow diverter devices, single antiplatelet therapy, intracranial aneurysms, hemorrhagic complications, thromboembolic complications

## Abstract

**Background:**

Hemorrhagic and thromboembolic complications (TECs) are the main concerns in the endovascular treatment of intracranial aneurysms using flow diverter devices (FDs). The clinical demand for single antiplatelet therapy (SAPT) is increasing especially with the development of devices with lower thrombogenicity profile. However, the safety of SAPT is not well established.

**Objective:**

To analyze the safety and efficacy of SAPT in terms of ischemic and hemorrhagic complications in patients undergoing FDs treatment for cerebral aneurysms.

**Methods:**

A systematic literature search and meta-analysis were conducted in PubMed, Ovid MEDLINE, Ovid Embase, and Web of Science from January 2010 until October 2022. Twelve articles which reported SAPT and data on hemorrhagic, TECs, and mortality following FDs treatment were included.

**Results:**

Overall, the 12 studies involved 237 patients with 295 aneurysms. Five investigated the safety and efficacy of SAPT in 202 unruptured aneurysms. Six studies focused on 57 ruptured aneurysms. One study included both ruptured and unruptured aneurysms. Among the 237 patients, prasugrel was most often used as SAPT in 168 cases (70.9%), followed by aspirin in 42 (17.7%) patients, and by ticagrelor in 27 (11.4%). Overall, the hemorrhagic complication rate was 0.1% (95% CI 0% to 1.8%). The TEC rate was 7.6% (95% CI 1.7% to 16.1%). In the subgroup analysis, the TEC rates of prasugrel monotherapy of 2.4% (95% CI 0% to 9.3%) and ticagrelor monotherapy of 4.2% (95% CI 0.1% to 21.1%) were lower than of aspirin monotherapy 20.2% (95% CI 5.9% to 38.6%). The overall mortality rate was 1.3% (95% CI 0% to 6.1%).

**Conclusion:**

According to the available data, SAPT regimen in patients undergoing FDs treatment for cerebral aneurysms has an acceptable safety profile, especially with the use of ADP-receptor antagonists.

## Introduction

Endovascular treatment using flow diverter devices (FDs) has revolutionized the treatment of intracranial aneurysms.^1,2^ During the past decade, the use and indications continued to expand from unruptured aneurysms to selected cases of ruptured aneurysm treatment.^3–7^ In addition to improving structural design aiming to increase efficacy, efforts are focusing to reduce the thrombogenicity of the devices. As such, different technological solutions are applied to achieve less thrombogenic metallic surface of the available FDs.^8–11^

Thromboembolic complications (TECs) are the main concern following FDs treatment. Dual antiplatelet therapy (DAPT) with aspirin and clopidogrel remains the primary regimen for their prevention.^12,13^ Some studies showed that DAPT may be associated with an increased hemorrhagic complication rate, especially in patients with acutely ruptured aneurysms.^14,15^ Recently, FDs with modification of the bare metal surface have been introduced, such as the pipeline embolization device (PED) with Shield technology (Medtronic Neurovascular, Irvine, California, USA) with integrated synthetic phosphorylcholine (PC) to the surface of the braid, or the p48 MW HPC or p64 MW HPC device with hydrophilic polymer coating (pHPC; Phenox, Bochum, Germany). Although these devices are still used under DAPT in the majority of cases, they may have the potential to be used with single antiplatelet therapy (SAPT), especially in specific conditions, such as in acute subarachnoid hemorrhage (SAH). As such, the risk of hemorrhagic complications could be theoretically reduced and eventually higher aneurysm occlusion rates may be achieved due to less aggressive antithrombotic effects. SAPT with such devices has however not yet been scientifically validated and remains experimental, as compassionate use or in the frames of clinical studies.^16,17^ In this systematic review and meta-analysis, we aimed to summarize the current practice and identify the safety and efficacy of different SAPT regimens in patients undergoing FDs treatment for cerebral aneurysms. This should help in designing clinical trials allowing for higher level evidence for SAPT usage.

## Methods

### Study selection and eligibility criteria

This systematic review and meta-analysis were performed in accordance with PRISMA guidelines.^18^ A systematic electronic search was done on PubMed, Ovid MEDLINE, Ovid Embase, and Web of Science from January 2010 until October 2022. The following keywords were used in the search using the Boolean operators OR and AND: “flow diverter,” “flow diversion,” “flow diverting,” “single antiplatelet,” “prasugrel,” “aspirin,” “acetylsalicylic acid,” “clopidogrel,” and “ticagrelor.” Two authors (ZK, YHM) independently performed the full-text screening. The inclusion criteria were as follows: English language, single antiplatelet therapy, intracranial aneurysm treated with FDs and data availability on periprocedural and postoperative complications. Exclusion criteria included case reports, case series with fewer than five patients, review articles, guidelines, technical notes, and in vitro studies.

### Outcome measures

Epidemiologic data included the number of patients, rupture status, hemorrhagic and ischemic complications, and mortality. TECs were defined as any symptomatic or asymptomatic thromboembolic events, and cerebral infarcts associated with FD treatment. Hemorrhagic complications were defined as subarachnoid or intracerebral hemorrhage. Asymptomatic perforator occlusions and complications caused by intraoperative technical error were not included.

### Statistical analysis

Event rate and 95% CI for each outcome were assessed according to the exposure variables. Statistical tests were two-sided, and *p*-values < 0.05 were considered significant. The I^2^ statistic was used to estimate heterogeneity across the included studies and >50% was considered high heterogeneity. All analyses were conducted using STATA version 15.

## Results

**Figure 1.**
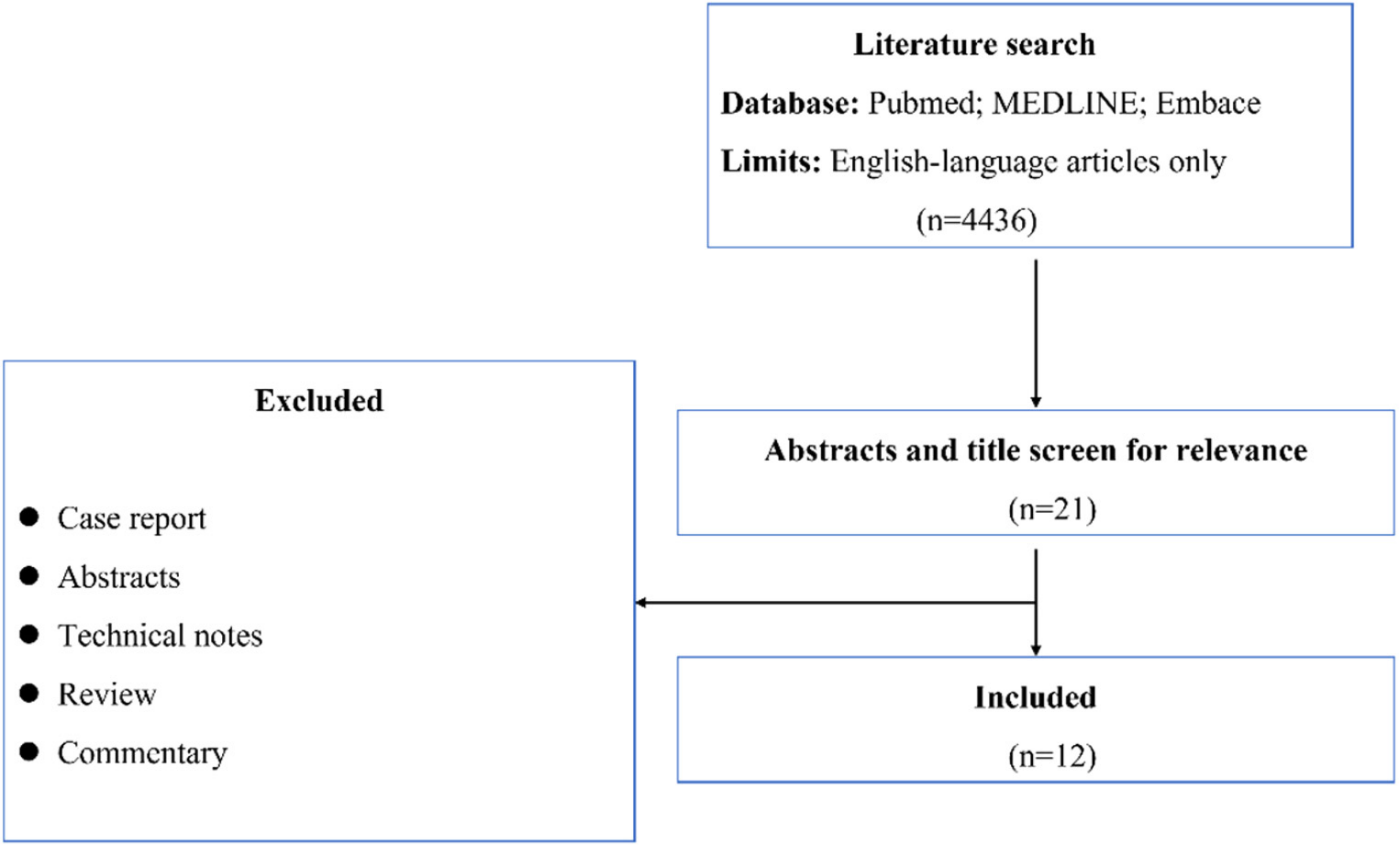
Screening and selection process of studies

A total of 4436 studies were identified during the initial search. Of these, 12 studies met our inclusion criteria (Figure 1). There were 11 retrospective studies and one prospective study (Table 1). Overall, these studies involved 237 patients with 295 aneurysms. Five studies investigated the safety and efficacy of SAPT in 202 unruptured aneurysms.^16,19–22^ Six studies focused on 57 ruptured aneurysms.^17,23–27^ One study included both ruptured and unruptured aneurysms.^28^ Of the 12 studies, 10 studies with 207 patients used surface-modified flow diverters. One study only used the bare metal PED device. In one study, both bare-metal and surface-modified flow diverters were used in conjunction with SAPT.^24^ Concerning the type of SAPT, prasugrel monotherapy was used in five,^16,20–22,26^ aspirin in three,^17,19,25^ and ticagrelor in one study.^28^ In three studies different antiplatelet drug regimens were used for SAPT.^23,24,27^ Among the 237 patients, 168 (70.9%) received prasugrel, 42 (17.7%) received aspirin, and 27 (11.4%) received ticagrelor. The overall mortality was 3.4% and the overall major stroke rate was 5.9%. In 11 studies, occlusion classification were recorded and complete aneurysm occlusion rate was 69.8%.

**Table 1.** Summary of studies.

Study	Patients	Aneurymsm	Unruptured	Device	Drug	Hemorragic complications	Thrombotic complications	Mortality
Lobsien^23^	10	13	No	pHPC	Aspirin/Ticagrelor	0	1	1
Luis^19^, 2021	7	8	Yes	pHPC	Aspirin	0	3	0
Bhogal1^6^, 2022	24	30	Yes	pHPC	Prasugrel	0	0	0
Tanburoglu^24^	6	6	No	SURPASS/PED/FRED	Ticagrelor/Prasugrel	0	0	1
Guzzardi^25^	7	7	No	pHPC	Aspirin	0	1	0
Mahmoud^28^	24	36	Yes/No	PED	Ticagrelor	1	1	1
Madjidyar^26^	9	9	No	PED	Prasugrel	1	0	1
Manning^17^	14	14	No	PED	Aspirin	2	2	3
Marta^27^	8	8	No	pHPC	Aspirin	0	4	0
Bhogal^20^, 2019	5	5	Yes	pHPC	Prasugrel	0	0	0
Luis^21^, 2021	21	27	Yes	pHPC	Prasugrel	0	4	0
Hellstern^22^	102	132	Yes	pHPC	Prasugrel	1	4	0

pHPC, p48/p64 MW flow modulation device; PED, pipeline embolization device; SURPASS, Surpass streamline stent; FRED, flow-redirection endoluminal device.

### Procedural outcomes

**Figure 2.**
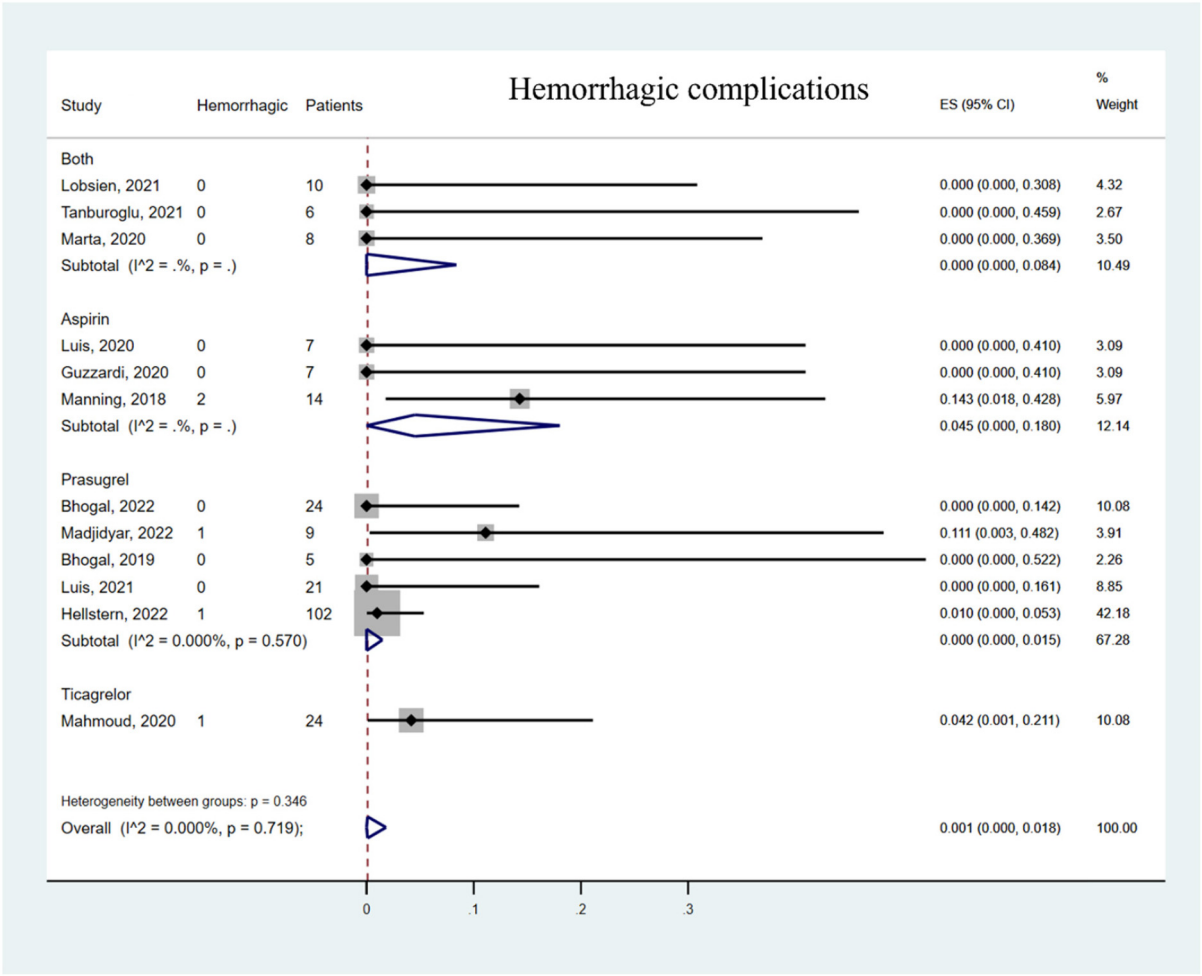
Forest plot of hemorrhagic complication rate. Meta-analysis for hemorrhagic complication rate and subgroup stratification by antiplatelet drug.

**Figure 3.**
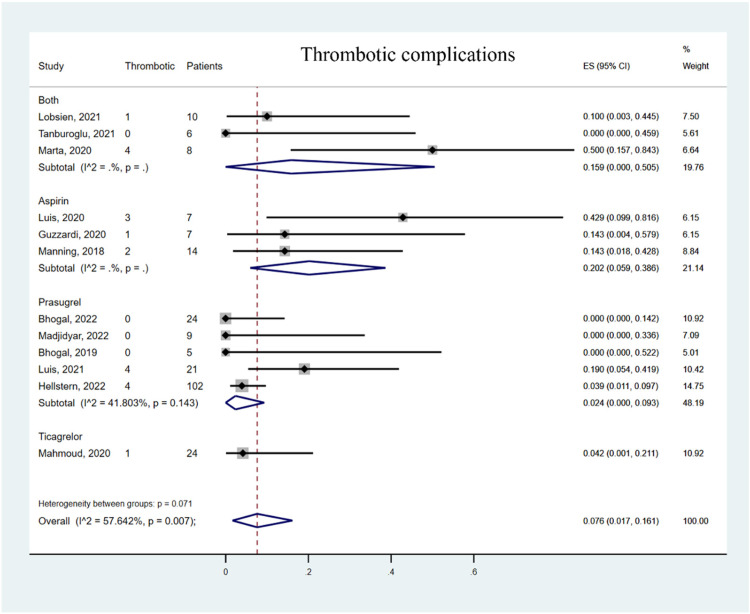
Forest plot of thrombotic complication rate. Meta-analysis for thrombotic complication rate and subgroup stratification by antiplatelet drug.

**Figure 4.**
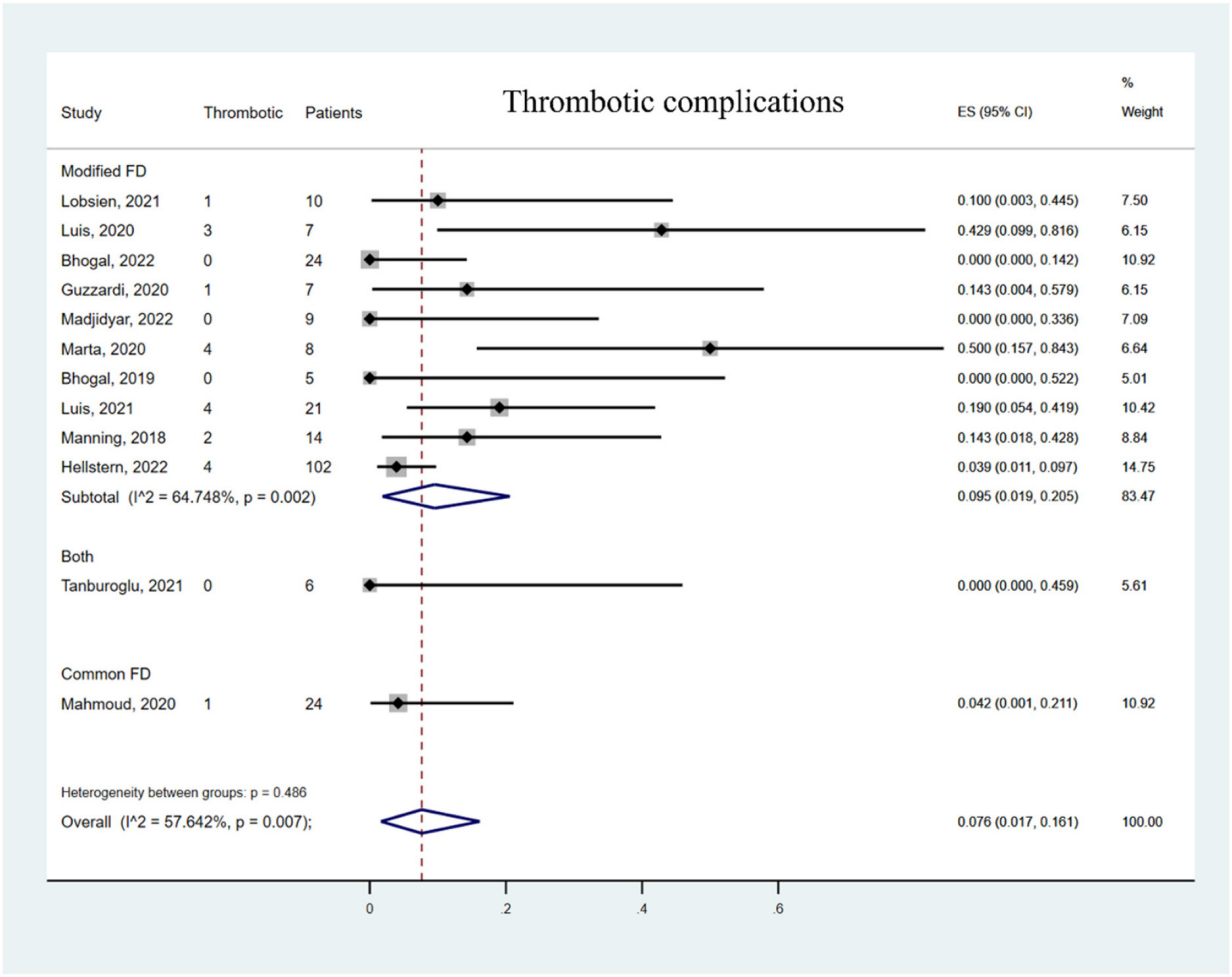
Forest plot of thrombotic complication rate. Meta-analysis for thrombotic complication rate and subgroup stratification by FD type. FD, flow diverter.

Overall, the hemorrhagic complication rate was 0.1% (95% CI 0% to 1.8%, Figure 2). The TEC rate was 7.6% (95% CI 1.7% to 16.1%, Figure 3). In the subgroup analysis, the hemorrhagic complication rate was lowest with prasugrel monotherapy 0% (95% CI 0% to 1.5%) compared to aspirin monotherapy (4.5%, 95% CI 0% to 18%) and ticagrelor monotherapy (4.2%, 95% CI 0.1% to 21.1%). The TEC rates of prasugrel monotherapy 2.4% (95% CI 0% to 9.3%) and ticagrelor monotherapy 4.2% (95% CI 0.1% to 21.1%) were lower than aspirin monotherapy 20.2% (95% CI 5.9% to 38.6%). The surface-modified FD subgroup showed higher TEC rates 9.5% (95% CI 1.9% to 20.5%) compared with common FD subgroup 4.2% (95% CI 0.1% to 21.1%, Figure 4), possibly related to the significant heterogeneity in the studies, especially in the surface modified FD subgroup (I^2^  =  64.748%, *p*  =  0.002).

**Figure 5.**
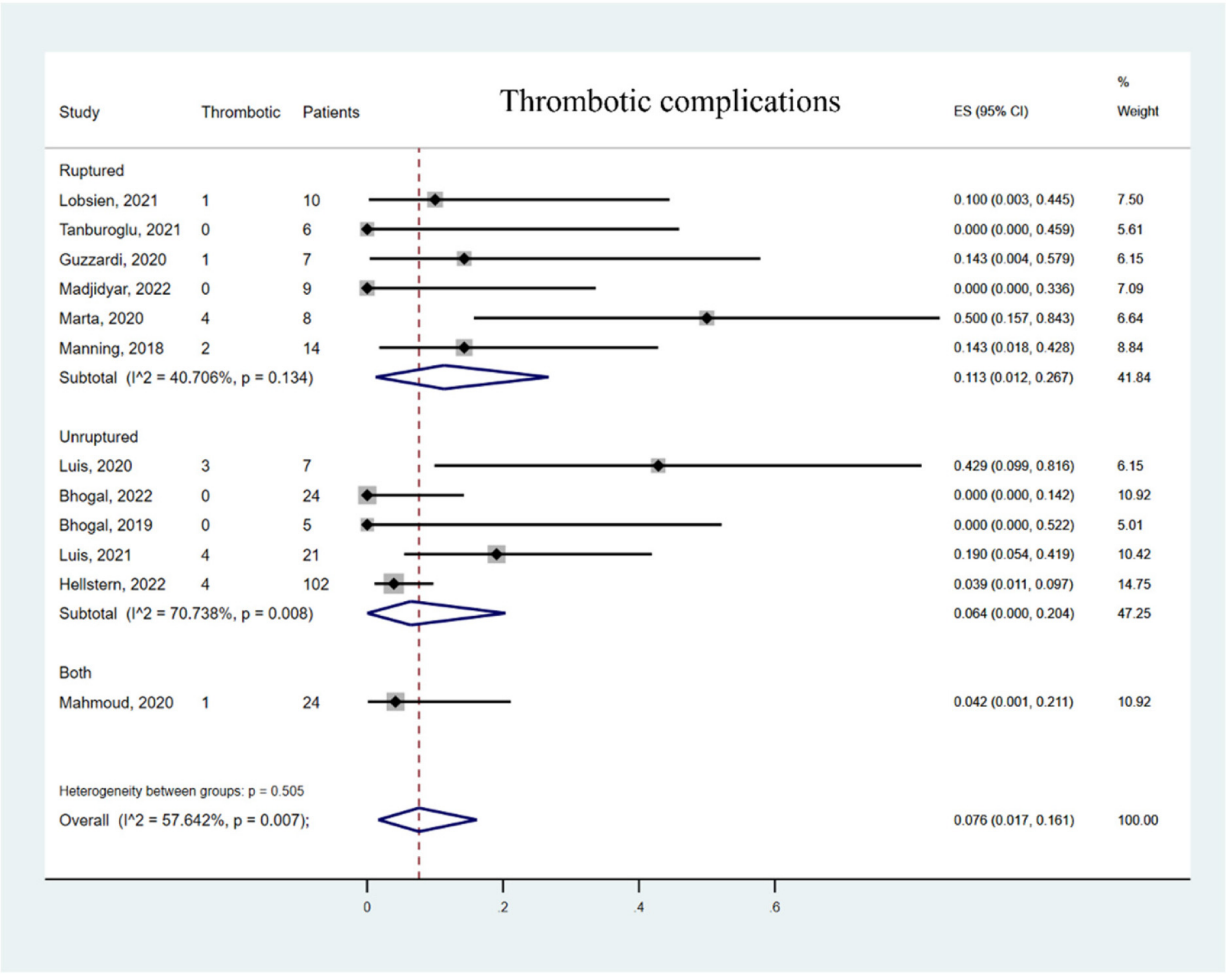
Forest plot of thrombotic complication rate. Meta-analysis for thrombotic complication rate and subgroup stratification by aneurysm rupture status.

**Figure 6.**
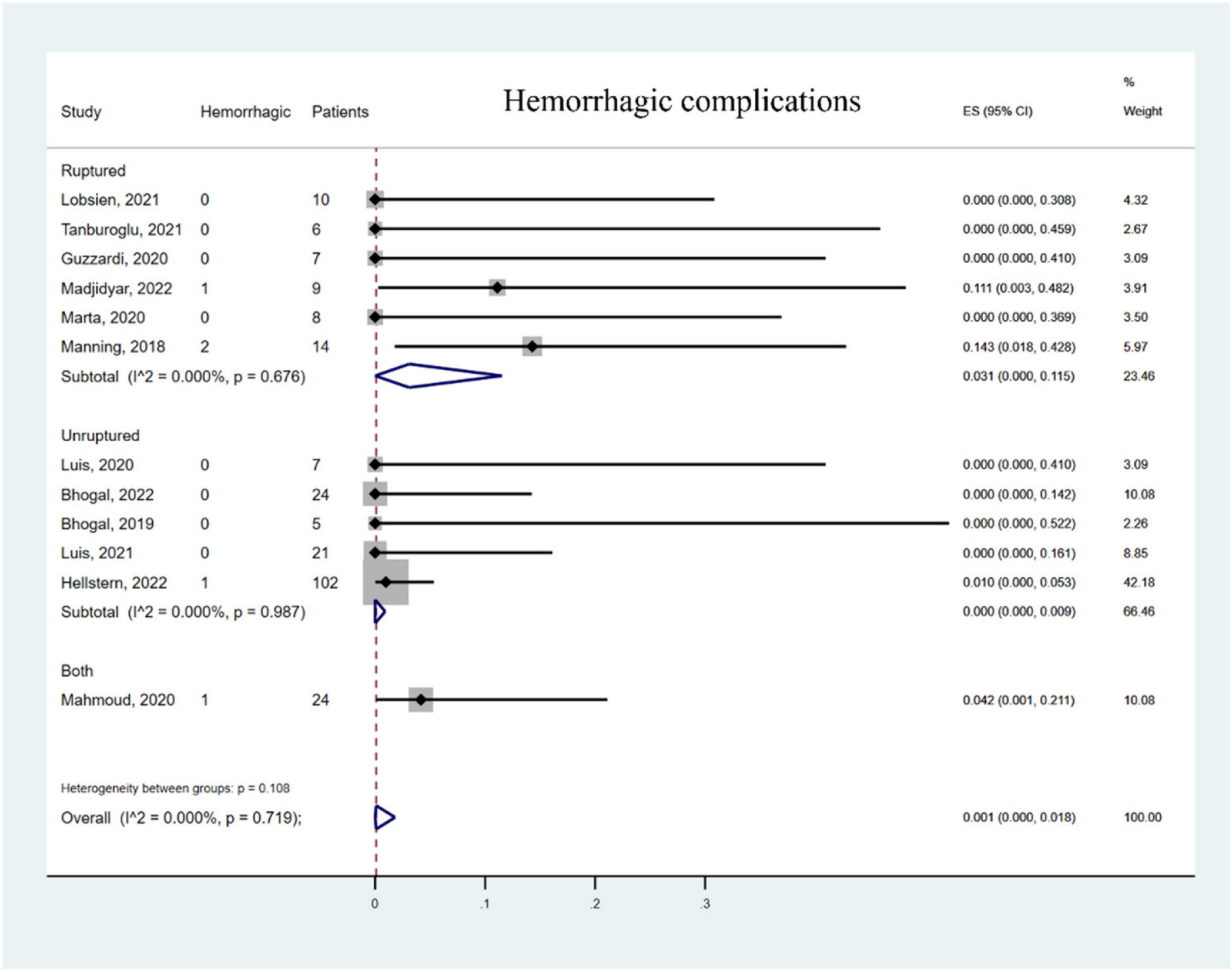
orest plot of hemorrhagic complication rate. Meta-analysis for hemorrhagic complication rate and subgroup stratification by aneurysm rupture status.

**Figure 7.**
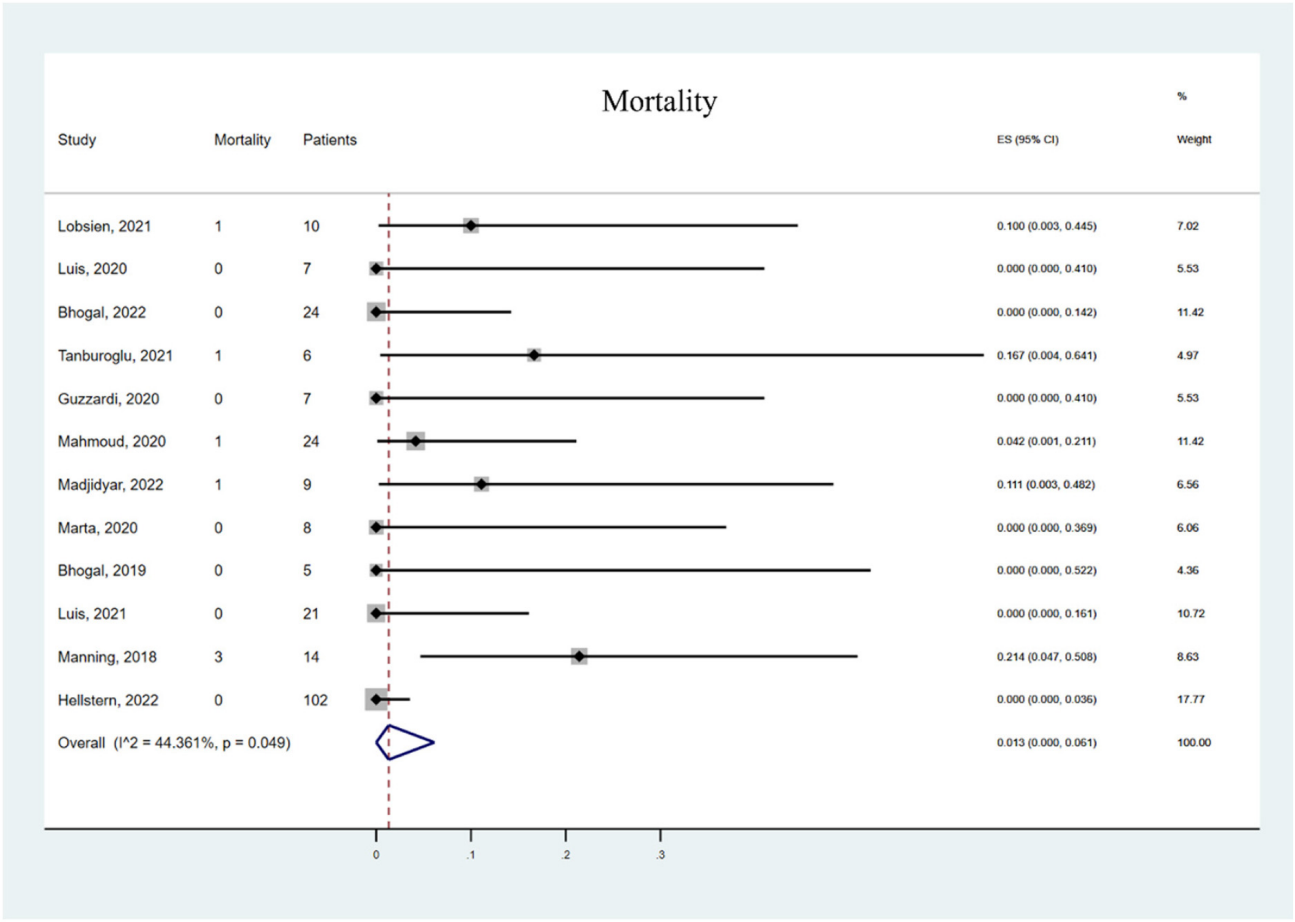
Forest plot of mortality rate.

The ruptured intracranial aneurysm (IA) subgroup showed a higher TEC rate 11.3% (95% CI 1.2% to 26.7%) compared to the unruptured IA subgroup 6.4% (95% CI 0% to 20.4%, Figure 5). There was significant heterogeneity in the unruptured IA subgroup (I^2^  =  70.738%, *p*  =  0.008). The ruptured IA subgroup also showed higher hemorrhagic complication rates (3.1%, 95% CI 0% to 11.5%) compared with the unruptured IA subgroup (0%, 95% CI 0% to 0.9%, Figure 6). This indicated that there was heterogeneity among these studies. Furthermore, compared with other studies, 2 studies with 15 patients reported an unusually high TEC rate. The overall mortality rate in the 12 studies was 1.3% (95% CI 0% to 6.1%, Figure 7).

## Discussion

This systematic review and meta-analysis have found that there are only a few articles that discuss the use of SAPT in the treatment of cerebral aneurysms. However, the study has also shown that the safety of SAPT depends largely on the medication regimen used, with prasugrel having the lowest rates of complications.

In recent years, several studies have investigated the effect of antiplatelet therapy on hemorrhagic and thrombotic complications in patients with cerebral aneurysms treated with FD,^29–31^ and DAPT with aspirin and clopidogrel being still the most often used medication for this purpose. However, the optimal dose and duration of post-treatment antiplatelet regimen is not standardized. In order to assess the risk of perioperative thrombotic and hemorrhagic events and find optimal dose and duration of antiplatelet regimen, platelet function testing, particularly the Verifynow Platelet reactivity Unit (PRU) assay has been adopted and shown some predictive value for these events.^32,33^ Almandoz et al. reported that a last-recorded P2Y12 reaction units value of <60 or >240 was the only independent predictor of all and major thromboembolic and hemorrhagic complications up to 6 months after PED procedures.^34^ In a retrospective study, Bhogal et al. evaluated the safety and efficacy in patients under SAPT with prasugrel.^16^ In that study, platelet inhibition level was tested and adequate response was defined as PRU < 100. In an earlier systematic review to investigate the risk of ischemic and hemorrhagic complications associated with common antiplatelet regimens following PEDs treatment, Saber et al. reported that DAPT with high-dose aspirin (>150 mg) and clopidogrel for at least 6 months was associated with a reduced incidence of ischemic events, without affecting the risk of hemorrhagic events. Overall, the hemorrhagic complication rate was 5% (95% CI 4% to 6%), and the ischemic complication rate was 7% (95% CI 6% to 9%).^35^

A number of new, potentially hypothrombogenic surface FDs such as the Pipeline shield, p48 Flow Device HPC and Derivo Embolization Device (DED) have been used to treat intracranial aneurysms.^36–38^ Yeomans et al. prospectively evaluated the occlusion rate and clinical outcomes following endovascular treatment of cerebral aneurysms with Pipeline Shield.^39^ They reported a periprocedural complication rate of 6.2% and there were no post-procedural complications. Furthermore, at 6 months post-procedure, satisfactory occlusion (defined as Raymond–Roy occlusion classification 1 or 2) was achieved in 90.6% and 93.8% of patients by way of magnetic resonance angiography and digital subtraction angiography assessment, respectively. Trivelato et al. investigated the safety and efficacy of the DED for emergency treatment of ruptured intracranial aneurysms.^40^ In this multicenter, prospective, and single-arm trial, 146 patients with 183 intracranial aneurysms were treated. They reported that endovascular treatment of intracranial aneurysms with the DED was a safe and effective treatment and was not associated with serious adverse events. Although DAPT is still the main choice for FDs, a limited number of studies have evaluated the safety and efficacy of SAPT such as aspirin, ticagrelor, or prasugrel for FDs.^21,28,41^

In our analysis, the total rate of hemorrhagic complications was 0.1%, which is lower than that with DAPT reported in recent studies.^30,42,43^ Park et al. evaluated the efficacy and safety of DAPT with ticagrelor and aspirin versus DAPT with clopidogrel and aspirin for stent-assisted coiling or FD.^31^ The rate of hemorrhagic complications was 9.2% in the clopidogrel group and 6.5% in the ticagrelor group. In that study, the higher hemorrhagic complication rate may be due to the size, location, and shape of the aneurysm. Although FD implantation under SAPT raises concerns regarding TECs, the total rate of TECs in our analysis was 7.6%, which is similar to that with DAPT. According to our meta-analysis and somewhat unexpected, the surface-modified FD subgroup showed higher TEC rates compared with common FD subgroup. One reason for this interesting result may be related to the more frequent use of aspirin as monotherapy in the surface-modified FD subgroup, with demonstrated higher TEC rates. Another explanation may be related to the lower number of patients treated with bare metal FDs, leading to bias. Saber et al. performed a meta-analysis investigating the risk of ischemic and hemorrhagic complications associated with DAPT using clopidogrel and aspirin following FD treatment.^35^ They found that the ischemic complication rate was 7%. Our meta-analysis showed similar TECs and lower HCs compared to DAPT.

### Aspirin monotherapy

In the three studies with 28 patients included in this meta-analysis investigating the safety and efficacy of FD under aspirin monotherapy, the overall TEC rate was 20.2%. In the pilot study, seven patients were treated with the p48 MW HPC for distal intracranial unruptured aneurysms.^19^ All patients received aspirin 100 mg/day for 7 days before treatment, and the rate of TECs was 42.9%. Lobsien et al. reported their retrospective, multicenter series of 13 ruptured aneurysms treated with either the p64MW HPC or p48MW HPC under aspirin/prasugrel monotherapy.^23^ Eight patients received aspirin at a dose of at least 250 mg/ day. The TEC rate was 10%. In contrast, Giuseppe et al. retrospectively evaluated seven patients treated for acutely ruptured aneurysms with a p48 MW HPC or p64 MW HPC.^25^ The TEC rate was 14.3%. These seven patients received aspirin monotherapy with a loading dose (500 mg) at the beginning of the procedure. After treatment, all patients started SAPT with twice-daily doses of 300 mg aspirin for 1 week, which was then reduced to twice-daily doses of 100 mg at 1-month angiographic evaluation, and subsequently reduced to a daily dose of 100 mg.

### Prasugrel monotherapy

Five studies with 161 patients investigated the outcome of FDs under prasugrel monotherapy. The overall TEC rate was 2.4%. The highest rate of TEC was 19% observed in a prospective and single-arm study.^21^ In the prospective study by Luis et al., 21 patients with 27 distal aneurysms were treated with the p48 MW HPC for distal intracranial unruptured aneurysms. All patients received prasugrel 5 mg or 10 mg/daily for 7 days before treatment until 6 months after treatment. In a recent retrospective study, Hellstern et al. evaluated 102 patients with 132 intracranial aneurysms treated with p64 MW HPC.^22^ The TEC rate was 3.9%. All patients received a loading dose of 30 mg prasugrel as SAPT at least 3 days prior to the procedure followed by doses of 10 mg/day for 6 months. Pervinder et al. retrospectively evaluated 24 patients with 30 unruptured aneurysms treated with p48 MW HPC or p64 MW HPC under prasugrel monotherapy and they did not record TECs.^16^ The premedication regimen consisted of 10 mg of prasugrel PO daily for at least 5 days pre-operatively. In all patients, prasugrel was continued for 6 months and then converted to aspirin 100 mg/day for at least 2 years. The meta-analysis showed a good efficacy of prasugrel for the prevention of TECs following FDs.

Our meta-analysis has potential limitations that need to be considered when interpreting the results. The limited data are based on observational retrospective or prospective studies, which may have the risk of both selection and publication bias. In addition, the outcome and complication rates were heterogeneous due to various antiplatelet drugs and regimens, aneurysm status, dosage, and duration of DAPT between studies. Furthermore, the antiplatelet protocol with respect to period, dosage and patient characteristics, has not been standardized. Still, these data may provide additional guidance for future studies.

## Conclusion

This systematic review and meta-analysis demonstrated that SAPT with prasugrel had the best safety profile in the treatment of intracranial aneurysms with FDs. These data should be used for designing future studies of DAPT in FD treatment of cerebral aneurysms.
